# Use of multikinase inhibitors/lenvatinib concomitant with locoregional therapies for the treatment of radioiodine‐refractory differentiated thyroid cancer

**DOI:** 10.1002/cam4.5108

**Published:** 2022-10-06

**Authors:** Miguel‐Ángel Berciano‐Guerrero

**Affiliations:** ^1^ Medical Oncology Intercenter Unit Regional and Virgen de la Victoria University Hospitals Málaga Spain

**Keywords:** enolization, lenvatinib, radiotherapy, surgery

## Abstract

Locoregional recurrence of differentiated thyroid cancer (DTC) occurs in 20% of thyroid cancer patients. Currently, there are many strategies for management of locoregional recurrence of DTC that lead to local control of the disease. The introduction of lenvatinib into the therapeutic armamentarium provides a new option for the treatment of radioiodine‐refractory DTC (RR‐DTC). However, results for simultaneous treatment with lenvatinib and locoregional therapies are unknown in patients with RR‐DTC. This paper reviews the current status of this approach and gives recommendations on the management of lenvatinib during concomitant locoregional procedures.

## Abstract 2

Locoregional recurrence of differentiated thyroid cancer occurs in 20% of thyroid cancer patients. Strategies for management of recurrence of radioiodine‐refractory differentiated thyroid cancer include lenvatinib. This paper reviews simultaneous treatment with lenvatinib and locoregional therapies.

## 1 INTRODUCTION

Locoregional recurrence of differentiated thyroid cancer (DTC) occurs in 20% of thyroid cancer patients.^1^ For this reason, multidisciplinary tumor boards have been created to manage DTC in a multimodal manner. This has enabled different therapeutic strategies based on local treatments such as surgery, enolization, embolization, or radiotherapy to be used primarily in early‐stage DTC aiming for cure of the disease.^2^

The objective of this paper is to review the available literature on the use of various locoregional techniques for management of advanced radioiodine‐refractory DTC (RR‐DTC) in the absence of clinical trials that combine these techniques with multikinase inhibitors (MKIs).

## 2 HISTORICAL PERSPECTIVE

Several years ago, new MKIs were introduced for the treatment of RR‐DTC and have become the current standard in this clinical scenario. Because lenvatinib therapy is active in patients with locally advanced thyroid cancers not operable due to high risk of perioperative complications (including in those naïve to radioactive iodine), some patients may benefit from modifications in the treatment sequence.^3^, ^4^ The most likely situation involves a need for surgical intervention while receiving systemic therapy. Specifically, ‘oligoprogression’ may happen in some tumors with insidious growth leading to metastases progressing at different rates.^5^ In this context, use of locoregional techniques concomitant with MKI treatment could be the best option to manage RR‐DTC presenting with this heterogeneous behavior. However, there are scarce published data for this approach in thyroid cancer, primarily because of the relatively short time since the approval of MKIs in this scenario and the heterogeneity of the clinical presentations. The objective of this review is to clarify the published data to facilitate the management of patients with RR‐DTC.

## 3 CURRENT SITUATION AND TREATMENT RECOMMENDATIONS

The different approaches to treatment of RR‐DTC that can be considered are described below.

### 3.1 Surgery

Use of surgery in disseminated disease is unusual, although it may be considered in circumstances in which the systemic disease is controlled or has achieved a response and, as mentioned before, there is oligoprogression. Having said this, given that the functional integrity of the neck is difficult to preserve with multiple surgeries, it is recommended to manage MKI‐unresponsive disease mainly in anatomic locations other than the neck. Thus, surgery may be considered for patients with single distant metastases, including patients with a single bone metastasis, brain metastases, or limited pulmonary metastases. The 5‐year survival for 31 patients with papillary cancer after thoracic metastasectomy was 64%, and radical surgical extirpation of isolated bone metastases is associated with improved survival.^1^

Surgery has been proved helpful in the management of DTC in conjunction with lenvatinib, with excellent results.^6^, ^7^, ^8^, ^9^ However, it is worth mentioning that impaired wound healing has been reported in patients receiving lenvatinib despite no formal research on this phenomenon having been done to date.^10^ Therefore, temporary interruption of lenvatinib treatment should be considered for patients undergoing major surgical procedures. Based on its half‐life of 28 hours measured in healthy volunteers, recommendations made in a phase III trial of patients with advanced renal cell carcinoma (aRCC) likely to receive surgery during the study, can be extrapolated to the thyroid cancer population.^11^ In addition, a real‐life study has recently been published from a major Japanese hospital, where Toda et al show that their experience with invasive procedures in 14 patients treated with lenvatinib is consistent with the following recommendations^12^:

- For minor procedures: stop lenvatinib at least 2 days before the surgery and restart it at least 2 days after, once there is evidence of adequate healing and no risk of bleeding.
- For major procedures: stop lenvatinib at least 1 week (5 half‐lives) prior to surgery and then restart it at least 1 week after, once there is evidence of adequate healing and no risk of bleeding.

Caution is advised as these recommendations were made for patients with aRCC receiving lenvatinib.^11^ However, they are likely to be applicable to patients with normal renal and hepatic function, thus with expected normal drug clearance. Despite the scarcity of robust clinical data, surgery with concomitant lenvatinib is a possible option to manage RR‐DTC patients.

A promising and possible strategy reported by several clinical cases^13^ and that is being tested in several clinical trials (Table 1) is the use of MKIs for neoadjuvant treatment of thyroid cancer. However, more robust data are needed for standardization in clinical practice. In that case, it will be necessary to determine which are the appropriate candidates and which tumor factors benefit most from said strategy.

**TABLE 1:** Clinical trials with tyrosine kinase inhibitors and local interventions in differentiated thyroid cancer

Recruting clinical trials using locoregional approaches for Thyroid Cancer									
Clinical trial number	Title	Phase	*N*	Type of Cancer	Locoregional Intervention	Locoregional treatment and MKi	MKi	Outcomes measures	Locations (countries)
NCT03975231	Dabrafenib, Trametinib, and IMRT in Treating Patients With BRAF Mutated Anaplastic Thyroid Cancer	I	20	Anaplastic Thyroid Cancer	Radiation: Intensity‐Modulated Radiation Therapy (IMRT)	Concurrent	Dabrafenib Trametinib	Maximum tolerated dose of combination therapy of dabrafenib and trametinib administered concurrently with intensity‐modulated radiation therapy (IMRT) Objective response rate Time to progression for local disease recurrence Overall survival Progression free survival	USA
NCT04321954	Lenvatinib in Locally Advanced Invasive Thyroid Cancer	II	30	Any thyroid cancer (excluded medullary or anapestic thyroid cancer)	Procedure: Therapeutic Conventional Surgery	Neoadjuvant	Lenvatinib	Overall R0/R1 resection rate Resection rate of R0 Resection rate of R1 Change in Surgical complexity and morbidity score (SCMS) Primary surgery response rate Number of Participants with Treatment Related Adverse Events as Assessed by CTCAE v 5.0 Unresectable to resectable conversion rate	USA
NCT04759911	Selpercatinib Before Surgery for the Treatment of RET‐Altered Thyroid Cancer	II	30	Medullary Thyroid Cancer	Procedure: Therapeutic Conventional Surgery	Neoadjuvant	Selpercatinib	Objective response rate (ORR) Tumor response R0/R1 resection rates Progression free survival (PFS) Locoregional PFS Surgical morbidity/ complexity score Overall survival (OS) Incidence of adverse events Quality of life Patient‐reported outcome	USA
NCT04739566	Dabrafenib and Trametinib Combination as a Neoadjuvant Strategy in BRAF‐positive Anaplastic Thyroid Cancer	II	18	Anaplastic Thyroid Cancer	Procedure: Conventional Surgery	Neoadjuvant	Dabrafenib Trametinib	Overall response rate (ORR) Number of R0 resections after 3 months of neoadjuvant combination therapy with anti‐BRAF and MEK inhibitors. Safety Profile (Number / Severity of Serious Adverse Events, SAEs) Percentage of patients who received a complete response 3 months after the first dose of treatment. Health‐related quality of life Progression‐Free Survival (PFS) Overall Survival (OS)	Russian Federation
NCT04675710	Pembrolizumab, Dabrafenib, and Trametinib Before Surgery for the Treatment of BRAF‐ Mutated Anaplastic Thyroid Cancer	II	30	Anaplastic Thyroid Cancer	Procedure: Conventional Surgery Radiation: Intensity‐ Modulated Radiation Therapy	Neoadjuvant	Dabrafenib Trametinib Pembrolizumab	Complete gross surgical resection (R0 or R1 resection) Overall survival (OS) Tumor response Progression free survival (PFS) Surgical morbidity/ complexity Number of patients with adverse events as a measure of safety of neoadjuvant dabrafenib, trametinib, and pembrolizumab Number of patients with adverse events as a measure of safety of postoperative pembrolizumab plus IMRT Locoregional control Health related quality of life Patient‐reported symptoms	USA
NCT04693377	Cryoablation Combined With Stereotactic Body Radiation Therapy for the Treatment of Painful Bone Metastases, the CROME Trial	NA	40	Any cancer with low alpha/beta ratio (renal cell carcinoma, CRPC, sarcoma, thyroid cancer, CRC, melanoma)	Procedure: Cryosurgery Radiation: Stereotactic Body Radiation Therapy	Concurrent (allowed)	Any targeted therapy	Pain response Daily morphine equivalent (MEDD) Duration of response Local control Rate and severity of adverse and serious related adverse events Technical success for cryoablation	USA

Abbreviations: CRC: colorectal cancer; CRPC: castration‐resistant prostate cancer; N: Number of expected patients; NA: Not applicable; Number in clinicaltrials.gov; MKi: multikinase inhibitor.

### 3.2 Enolization

Enolization consists of percutaneous ethanol injection to limited cervical lymph node metastases. Long‐term local control can be achieved, and the technique is not associated with major complications. However, retreatment is sometimes necessary.^14^ As with surgery, there are no data available for combining this technique with MKI.

### 3.3 Radiofrequency ablation

Radiofrequency ablation (RFA) has been used primarily for liver tumors as an alternative to a surgical procedure. RFA of cervical, osseous, and pulmonary metastases is an alternative for patients who are poor surgical candidates and whose metastases do not concentrate radioiodine, but expertise in this treatment modality is not widely available.^15^ Furthermore, RFA can be used before surgery to improve surgical results and reduce morbidity and mortality.^16^ In the same way as other techniques, there are no data supported by clinical trials that generate sufficient scientific evidence. However, Porcelli et al, when reviewing other pathologies, conclude that it may be feasible to use these ablative techniques for the local control of oligoprogressive thyroid cancer.^17^

### 3.4 Embolization

Embolization has generally been used as adjuvant therapy to reduce surgical bleeding in patients with large or very vascular metastases.^18^ In addition, palliative embolization may reduce symptoms or be used prior to surgery. Its selective use for bone metastases may also be considered as palliative therapy because it reduces symptoms. However, embolization has also shown benefit in patients who have received concomitant treatment with radioiodine or radiotherapy.^19^, ^20^ There are no robust data on the use of embolization with MKI treatment, although Rodia et al report a study in which a patient is coadministered lenvatinib treatment without complications. In this case, the authors propose a reduction in the dose of lenvatinib in the period around the procedure.^21^

### 3.5 Radiotherapy

A recent in vitro study shows how MKIs and radiation significantly inhibit thyroid cancer growth by uptake of tyrosine kinase inhibitor,^22^ although the paucity of clinical studies leads to heterogeneity in treatment and recommendations. For patients with locally advanced unresectable disease, when radioiodine fails to control local growth and spread of disease, external beam radiotherapy is suggested for palliation. Radiation therapy in thyroid cancer has a palliative function for pain control or to control the local growth of lesions that cannot be approached with other techniques.^23^ The specific anatomic area and tumor volume should be considered prior to therapy. Whole brain radiotherapy in patients with brain metastases usually has no survival benefit,^24^ while resulting in neurological impairment in some patients. However, recent improvements in radiotherapy support that other techniques with less toxicity such as radiosurgery or tomotherapy can be considered.^25^

### 3.6 Lenvatinib

From its introduction, the treatment indications for lenvatinib have increased, possibly leading to its combination with locoregional therapies eventually opening new ways of combined thyroid cancer treatment.

Table 1 shows eight clinical trials that are currently recruiting patients and include locoregional strategies in the treatment of thyroid cancer. There are no results available yet.

**FIGURE 1:**
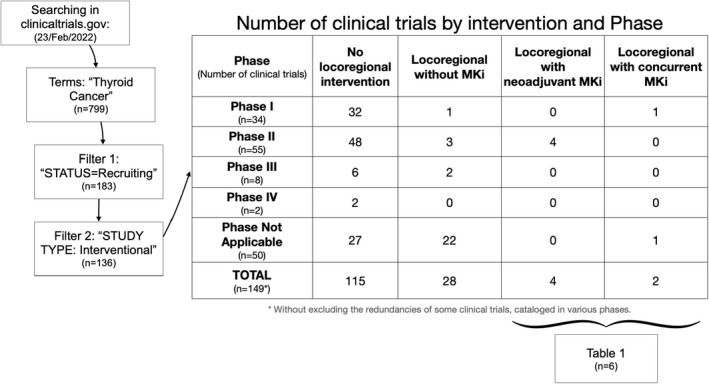
Systematic search of recruiting clinical trials in www.clinicaltrials.gov

Until there are sound published data to enable proper recommendations on the use of locoregional therapies during treatment with lenvatinib, assumptions can be made based on the previous recommendations specific for surgical procedures (Figure 1).

## 4 DISCUSSION

When treating a patient with oligometastatic progression of RR‐DTC, it is important to assess whether local techniques would provide benefit in terms of symptom control and can positively influence a longer survival time. As there is currently no solid evidence on the use of locoregional strategies in conjunction with lenvatinib or other MKIs, these questions remain unresolved. For the time being, decision‐making relies on the limited published real‐world experience and expert recommendations, while randomized clinical trials will provide stronger efficacy and safety data to better support clinical decisions in the future. This manuscript, in the absence of review articles in this field, aims to inform about the current state‐of‐the‐art treatment to facilitate management of thyroid cancer with MKI therapy.

## 5 CONCLUSION

The use of locoregional techniques during treatment with an MKI for thyroid cancer is feasible, although there is very little supportive scientific evidence to date. Overall, withdrawal of the MKI for a few days guarantees the absence of major complications, although it has also been proposed to reduce the dose during a specific window around the procedure. Currently, some clinical trials are under way that can provide more scientific evidence for the combination of locoregional techniques with an MKI.

## AUTHORS' CONTRIBUTIONS

Miguel Ángel Berciano Guerrero is the full author of the manuscript, as well as the tables and figures.

## ETHICS STATEMENT

Not applicable.

## FUNDING INFORMATION

The author received an honorarium payment from Eisai Farmacéutica SA in line with ICMJE guidelines.

## CONFLICT OF INTEREST

Dr Miguel‐Ángel Berciano‐Guerrero has received research funding, honoraria, and non‐financial or other support from BMS, MSD, Novartis, Pfizer, Pharmamar, Pierre‐Fabre, Roche and Sanofi and non‐financial or other support from Eisai Farmacéutica SA, BMS, MSD, Novartis, Pfizer, Pharmamar, Pierre‐Fabre, Roche and Sanofi.

## Data Availability

Not applicable.
